# Health Tract, No. 227

**Published:** 1865

**Authors:** 


HEALTH TRACT, No. 227.

### MEDICAL SCIENCE.

Aforetime it was a favorite saying that it needed only to have the "gift of gab," to make a good lawyer; to have power in the pulpit, a man must be thoroughly educated, have full faith in his doctrines, and a heart big with love for all of woman born; but to be a doctor, it required only to have a gold-headed cane, look wise, and say nothing. But times have changed; the people have become better informed, and the owl is no longer king, or high potentate. Many inquire with great earnestness, How does a doctor know what to do? and look at him at the bedside with a kind of wonderment and awe. The best method of becoming a successful practitioner is to cultivate the power of observation; but it must be done closely and correctly, and then, by the application of a sound judgment, the way to eminence is clear and certain. Many persons think that there is a great deal that is hap-hazard in the practice of medicine, but if they knew a little more, they would view it with admiration and with increasing interest, for the certainty and beauty of its operation. There are in the human system a number of "glands," which are really manufactories, workshops, or wheels, all of which must be kept in operation, or the man dies; the machinery of life stops as certainly as a clock ceases to tick, the moment one of its numerous wheels ceases to move. There is, however, this difference in the wonderful human machine: when one gland, or wheel, is out of order, some other gland or wheel will, for a while, act as a substitute, as if to give the ailing one a little time for rest; but if it does not, after a while, resume its operations, all the glands take alarm, and the machinery of life stops forever.

There are many beautiful illustrations of this fact, of constant occurrence, yet unobserved, except by the educated physician. The skin is a gland, so is the liver, and so the kidneys, manufacturing, respectively, "Perspiration," (sweat,) Bile and Urine. If while the skin is in active working order, delivering sweat-drops at every pore, these pores are closed suddenly, the wheels stop, the perspiration is "driven in," and must be worked out of the system by an increased amount of water thrown off through the kidneys or a sudden looseness of bowels; otherwise, a bad cold is the result and the water is passed off through the lungs in the shape of a thickish, pearly-looking phlegm or heavy, yellow matter. When the checking of perspiration, or the stopping of the working of a gland, is too sudden, death occurs in a few days, or even hours. Suppose, for example, the bowels are acting freely every few hours, and any thing is given to check them all at once, within the hour, a stroke of apoplexy in adults, and convulsions in children, sends them to the grave. It is the nature of opium in all its forms to stop the working of all glands in the system, hence it ought never to be given except to gain a little time to bring the system under the operation of other remedies which are radically curative. Opiates only alleviate; they smother, they hide the black spot on the wall like whitewash, but it is there still, it is only hidden; they eradicate, they cure nothing; their essential nature, their inevitable effect is to cause every gland in the system to work more and more sluggishly until the wheels of life move no more.

